# A wing expressed sequence tag resource for *Bicyclus anynana *butterflies, an evo-devo model

**DOI:** 10.1186/1471-2164-7-130

**Published:** 2006-05-31

**Authors:** Patrícia Beldade, Stephen Rudd, Jonathan D Gruber, Anthony D Long

**Affiliations:** 1 Department of Ecology and Evolutionary Biology, University of California at Irvine, Irvine, USA; 2 Institute of Biology of the University of Leiden, Leiden, The Netherlands; 3 Bioinformatics Laboratory, Turku Centre for Biotechnology, Turku, Finland

## Abstract

**Background:**

Butterfly wing color patterns are a key model for integrating evolutionary developmental biology and the study of adaptive morphological evolution. Yet, despite the biological, economical and educational value of butterflies they are still relatively under-represented in terms of available genomic resources. Here, we describe an Expression Sequence Tag (EST) project for *Bicyclus anynana *that has identified the largest available collection to date of expressed genes for any butterfly.

**Results:**

By targeting cDNAs from developing wings at the stages when pattern is specified, we biased gene discovery towards genes potentially involved in pattern formation. Assembly of 9,903 ESTs from a subtracted library allowed us to identify 4,251 genes of which 2,461 were annotated based on BLAST analyses against relevant gene collections. Gene prediction software identified 2,202 peptides, of which 215 longer than 100 amino acids had no homology to any known proteins and, thus, potentially represent novel or highly diverged butterfly genes. We combined gene and Single Nucleotide Polymorphism (SNP) identification by constructing cDNA libraries from pools of outbred individuals, and by sequencing clones from the 3' end to maximize alignment depth. Alignments of multi-member contigs allowed us to identify over 14,000 putative SNPs, with 316 genes having at least one high confidence double-hit SNP. We furthermore identified 320 microsatellites in transcribed genes that can potentially be used as genetic markers.

**Conclusion:**

Our project was designed to combine gene and sequence polymorphism discovery and has generated the largest gene collection available for any butterfly and many potential markers in expressed genes. These resources will be invaluable for exploring the potential of *B. anynana *in particular, and butterflies in general, as models in ecological, evolutionary, and developmental genetics.

## Background

The last decade has witnessed an increased effort at bringing together methods and ideas from evolutionary and developmental biology (evo-devo) to explore the genetic basis of morphological diversity [1-7]. Understanding the genetic bases of phenotypic variation is a fundamental challenge for evo-devo and for contemporary biology in general [8]. The color patterns on butterfly wings are a spectacular example of morphological variation, and have been established as an important system in the study of evolution and development [9-11]. These patterns, which vary both across and within species, are ecologically relevant and often have a known adaptive value, including amazing examples of mimicry [9,12-14] and adaptive phenotypic plasticity [15-17]. They have also been the object of developmental studies at different levels, including the genetic pathways involved in pattern formation, the physiological basis of pattern plasticity, and the biochemical pathways leading to pigment production (reviewed in [9,10]).

*Bicyclus anynana *has been established as a butterfly laboratory model which is well suited to address important questions in ecological, evolutionary and developmental genetics [9-11,18,19]. The study of *B. anynana *wing patterns has already made important contributions to evo-devo (reviewed in [10]). The patterns of phenotypic and genotypic variation in different eyespot traits have been explored within and across species [20-25], and a number of developmental pathways known from *D. melanogaster *wing development have been implicated in butterfly color pattern formation [15,26-29]. More recently, we have shown that candidate genes within these pathways can contribute to between-individual variation in wing pattern in *B. anynana *butterflies [19,30].

Knowledge from *D. melanogaster *wing development studies has undoubtedly greatly advanced our understanding of butterfly wing pattern formation. Yet, despite the power of this approach, it is limited in that it does not generate candidate genes outside known *Drosophila *wing genes. Lepidoptera (butterflies and moths) and Diptera are highly diverged and butterfly and dipteran wings are quite different (*e.g*., dipterans have a single pair of wings and no colored scales covering them). It thus seems unlikely that all the genes involved in butterfly wing pattern formation will be genes known from *Drosophila *wing development [19]. Clearly, a more unbiased search for pattern variation candidate genes is necessary to understand fully butterfly wing color evolution and development. Unfortunately, genomic resources in Lepidoptera are scarce, even though this order of holometabolous insects encompasses over 160,000 species, including many of economical importance (*e.g*., pollinators, agricultural pests, and silk producers). Most available information concerns the silkworm, *Bombyx mori *[31], with recently published expression sequence tag [32,33] and whole-genome shotgun [34,35] projects. Other moths (*e.g*., [36]) are not nearly as well studied and genomic resources for butterflies are scarcer still, despite ongoing expansion of gene collections for *Heliconius *[37]. Developing such resources is a fundamental step towards a detailed analysis of the genetic bases and consequences of some unusual biological properties of lepidopterans (*e.g*., heterogametic females, holocentric chromosomes, and derived wing color patterns), as well as those that distinguish butterflies from moths (*e.g*., diurnal life style and color vision [38]).

Important recent advances for *B. anynana *genomic resources include an ongoing project to build and analyze Bacterial Artificial Chromosome libraries, the development of germline transformation techniques to enable functional analysis of candidate genetic regions [39], and the characterization of 28 polymorphic microsatellites including a female-specific marker [40]. Until now, however, only twelve either partial or full-length coding sequences from *B. anynana *had been deposited in GenBank. As a way to identify a large number of genes for this species quickly and efficiently, we carried out an Expression Sequence Tag (EST) project. EST projects are a cost-effective and powerful way to identify a large number of expressed genes in any species [41,42], especially those without a genome project, and have been used in many different systems (*e.g*., [43-47]). Furthermore, many "downstream" applications stem from EST projects, including genome annotation, and the development of both high-density expression arrays, and gene-based genetic markers [42,48].

Here, we describe a *B. anynana *EST project in which we identify over 4,000 unique genes (or UniGenes) expressed in developing butterfly wings, together with DNA sequence polymorphisms in many of the more highly expressed transcripts. Our project was designed to maximize the likelihood of characterizing genes important in wing pattern formation and simultaneously identify polymorphisms in a fraction of these genes. The results described here characterize the most extensive EST/gene resource publicly available for any butterfly species. This collection will be the basis to build a gene-based linkage map and gene expression arrays for *B. anynana *butterflies, and will be of great utility in comparative genomics in the Lepidoptera.

## Results

### EST statistics

**Figure 1:**
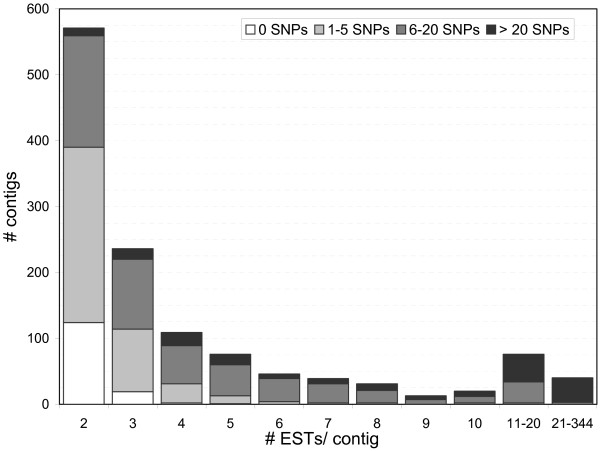
**Contig alignment depth and SNP identification**. Of the 4,251 UniGenes (contigs) identified, 2,994 were singletons (not shown) and all others had two or more ESTs (*i.e*., alignment depth of two or greater). The total number of contigs for each alignment depth class is represented by the height of the columns, and the different colors represent different SNP number classes. It should be noted that alignment depth here refers to the number of ESTs in each contig and does not necessarily imply a constant alignment depth at all sites along the contig sequence.

We processed and assembled a total of 10,159 ESTs from five different wing libraries (Table 1). These included 16 (~0.2%) of length shorter than 55 bp and 240 (~2%) which assembled into 166 UniGenes of low complexity (see Methods) and were excluded from further analysis (see Table 1). The other 9,903 EST sequences assembled into 4,251 UniGenes, of which 2,202 (~52%) were estimated to have coding potential using ESTscan (see Methods and Table 2). Our library subtraction procedure, based on removing ESTs observed to be at a high frequency in an initial sample of ~2,000 PCR-amplified inserts (see Methods), enabled us to obtain as many UniGenes with ~10,000 sequence reads as would have been obtained from ~23,000 clones without subtraction. Figure 1 illustrates EST redundancy in our project. About 70% (or 2,994) of the UniGenes we identified were singletons (and are not depicted in Figure 1), and 450 (or ~11%) resulted from aligning four or more ESTs. The maximum alignment depth observed was 344 ESTs in one contig. Multifasta files with the alignments for all multimember UniGenes are available from [49] as a single compressed file. Table S3 [see Additional file 3] lists all individual ESTs of length greater than 54 bp and related information, including the actual sequence (with letter case indicating PHRED quality; see Methods), sequence length, ratio of low quality nucleotides to total length, and the contig into which they were assembled. Table S4 [see Additional file 4] lists all contigs and all description and annotation information discussed in this report.

**Table 1:** cDNA libraries made from wing discs at different developmental stages

ID^a^	Nr of outbred individuals	Nr of ESTs^b^	GenBank Accession
5TH	35 caterpillars	2555 (83)	DY770898-DY773452
CPP	21 crawlers and 15 pre-pupae	1882 (61)	DY763294-DY765175
24H	33 females and 33 males	2014 (54)	DY765176-DY767189
48H	20 females and 20 males	1669 (26)	DY767190-DY768858
72H	10 females and 10 males	2039 (32)	DY768859-DY770897

^a ^See Methods for a full description of each library; ^b ^Total number of ESTs submitted to NCBI ESTdb and, in parentheses, the number of these which were excluded from the analysis (either shorter than 54 bp or part of the 166 UniGenes with low complexity scores; see Methods).

**Table 2:** General description of EST project sequences and assemblies

		length		
	# total	min	max	mean

ESTs (PHRED> = 20)	9903	55 (10)	1264 (1201)	502 (455)
contigs	4251	55	2763	583
peptides	2202	20	539	154

### Gene identification

**Figure 2:**
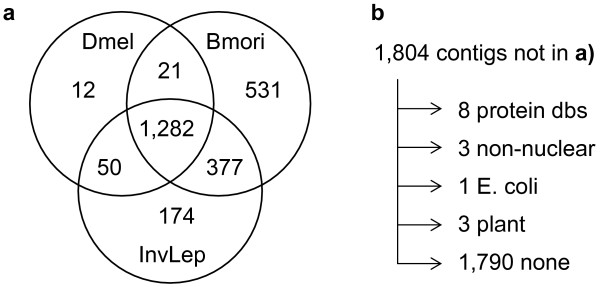
**UniGene identification through BLAST analyses toselected genomic collections**. **a) **Venn diagram summarizing gene identification based on BLAST against the genomic collections phylogenetically most relevant for *B. anynana*: "Dmel" for *D. melanogaster*, "Bmori" for the silkworm *B. mori*, and "InvLep" for lepidopteran nucleotide sequences and invertebrate proteins (see Methods and Table 3). About 42% of our 4,251 UniGenes did not have a significant BLAST hit for any of these three categories. **b) **Of the 1,804 genes not included in the Venn diagram, 14 showed a significant BLAST hit to at least one of the additional collections analyzed (details in Table 3 and in the text). The numbers on the left panel represent BLAST hits to groups of these collections (with some overlap across the collections).

Annotation of the *B. anynana *wing UniGenes was achieved through BLAST analyses against a number of different genomic collections. A total of 2,461 UniGenes (~58%) had a significant BLAST hit against at least one of the collections used in our analysis (Table 3; see Methods). The Venn diagram of Figure 2a illustrates UniGene annotation based on available genomic collections that are most relevant to this project: the model dipteran *D. melanogaster *and the silkworm *B. mori *(the lepidopteran species with most extensive published collection of genomic information), and diverse and dispersed collections of lepidopteran nucleotides and invertebrate proteins.

**Table 3:** Successful annotation based on BLAST against different gene collections

		BLAST hits		
Field	min E-value^a^	Total^b^	Best^c^	Unique^d^

Dmel.pro^1^	1e-171	1346	110.5	4
Dmel.nuc^1^	1e-147	264	7	6
Bmori.pro^2^	0e+00	1730	141.5	194
Bmori nuc^2^	0e+00	1265	1222	20
Bmori.wgs.nuc^2^	0e+00	1370	283	143
lep.nuc^3^	0e+00	1012	459.5	147
invert.pro^3^	1e-171	1488	170.5	4
NonRed.pro^4^	1e-175	1487	29	3
SwissP.pro^4^	1e-176	1136	25	1
organel.nuc^5^	3e-70	26	3	2
Rfam.nuc^5^	0e+00	27	2	0
plant.pro	1e-125	793	7.5	0
Ecoli.nuc	4e-12	7	1	1

^a ^The threshold maximum E-value was set to 1e-05. ^b ^Total number of contigs with significant E-value. ^c ^Number of contigs having the lowest E-value for each specified BLAST field. When the lowest and second lowest E-values were the same for a particular contig it counted as 0.5 for each of the hit fields. ^d ^Number of contigs having significant BLAST hits exclusively for one of the fields. Some BLAST fields were combined to give the counts in Figure 2: ^1 ^Dmel, ^2 ^Bmori, ^3 ^InvLep, ^4 ^protein databases, ^5 ^non-nuclear genes (as explained in the Methods). All collections used in our BLAST analysis were downloaded from public databases (see Methods) in June-August of 2005 (except for the organellar nucleotidic and the *E. coli *whole-genome sequences, both obtained in April 2004).

Even though all of the contigs included in Figure 2a showed a significant BLAST result against one of the invertebrate collections we defined (Dmel, Bmori, and InvLep; see Methods), the best BLAST hit was not always to one of these (Table 3). Fourteen UniGenes had best BLAST hits to non-nuclear or non-animal gene collections, and for six of these the ratio between E-values for the overall best BLAST hit and for the best hit to one of the invertebrate-specific genes (*cf*. Figure 2a) was greater than 100. These included three contigs that appeared to be either non-nuclear genes (lower hit against "organel.nuc"; contigs 3271, 3438) or non-coding RNAs (lower hit against "Rfam.nuc"; contig 1740), and three others likely corresponding to material from bacteria (lower hit against "Ecoli.nuc"; contig 847) or plants (lower hit against "plant.pro"; contigs 49 and 1172). Both contigs referred to above as "organel.nuc" actually correspond to chloroplast tRNA and thus add to the probable contaminants resulting from plant DNA incorporated in our libraries. This list of probable contaminants is likely to be an under-estimate of the genes that are not nuclear genes from butterfly wings. Our lepidopteran collection, for example, is an amalgam from many different independent studies deposited on EMBL in association to a lepidopteran species and does contain a number of organelle genes, non-coding RNAs, and potentially even bacteria and plant contaminants.

### Functional annotation

In order to assign putative functional roles to the identified UniGenes, we used the Gene Ontology (GO) annotations of *D. melanogaster *genes. Based on BLAST analysis against the *D. melanogaster *collections, we identified 1,249 *D. melanogaster *genes orthologous to at least one of our *B. anynana *transcripts. In addition, our BLAST analyses against the *B. mori *EST collections, in concert with our own annotation of the *Bombyx mori *collection via *D. melanogaster*, led to the identification of 240 additional *D. melanogaster *orthologs not identified by direct comparisons between *B. anynana *and *D. melanogaster *(see Methods). The list of 1,489 *B. anynana *UniGenes with *D. melanogaster *orthologs, and the complete collection of 14,187 *Drosophila *genes retrieved from FlyBase were used with GOminer [50] to assign GO terms to each *B. anynana *UniGene with a *D. melanogaster *ortholog and to look for GO categories enriched in our UniGene collection (cf. [51]; highlights in Table 4) [see Additional file 7 for a complete list]. GO annotations based on BLAST hits to the InterPro database are available in our web-accessible database in openSputnik [52].

**Table 4:** GO functional terms for *B. anynana *genes annotated to *Drosophila *CG numbers

GO TERMS	#	%	P-value
**Molecular function**			
Signal transducer activity (GO: 0004871)	72	7	0.3025
Structural molecule activity (GO: 0005198)	195	25	
Motor activity (GO: 0003774)	15	16	
Catalytic activity (GO: 0003824)	568	14	
Transporter activity (GO: 0005215)	151	12	0.3294
Binding (GO: 0005488)	651	15	
Antioxidant activity (GO: 0016209)	13	33	
Enzyme regulator activity (GO: 0030528)	52	14	
Transcription regulator activity (GO: 0030528)	103	12	0.0011
Transcription factor activity (GO: 0003700)	28	7	
Translation regulator activity (GO: 0045182)	26	28	
			
**Biological process**			
Development (GO: 0007275)	190	8	
Larval or pupal development (GO: 0002165)	57	10	
Pattern specification (GO: 0007389)	37	10	
Metamorphosis (GO: 0007552)	43	9	
Aging (GO: 0007568)	5	10	
Pigmentation (GO: 0048066)	4	7	
Regulation of development (GO: 0050793)	5	7	
Morphogenesis (GO: 0009653)	89	9	0.1183
Embryonic development (GO: 0009790)	46	9	
Physiological process (GO: 0007582)	1013	13	
Behavior (GO: 0007610)	31	8	
Cellular process (GO: 0009987)	1000	13	
Regulation of biological process (GO: 0050789)	217	13	
			
**Highlights**			
Structural constituent of ribosome (GO: 0003735)	110	57	
Tubulin (GO: 0045298)	4	25	
Wing disc development (GO: 0035220)	21	11	
Tracheal system development (GO: 0007424)	13	10	
*Wnt *receptor signaling pathway (GO: 0016055)	11	15	
*sevenless *signaling pathway (GO: 0045500)	2	22	
*Notch *signaling pathway (GO: 0007219)	3	7	
*frizzled *signaling pathway (GO: 0007222)	6	29	
Eye pigment metabolism (GO: 0042441)	4	13	

# is the number of *B. anynana *gene annotations obtained via BLAST analysis *D. melanogaster *and *B. mori *(total CG numbers in *B. anynana *with GO annotation = 1,164) that belong to the listed GO categories. % refers to the proportion of *D. melanogaster *genes (total CG numbers in *D. melanogaster *with GO annotation = 10,391) found in *B. anynana *for each GO category. The categories specified in this list are a limited subset of all GO categories found to be represented [see complete list in Additional file 7]. A Chi-square test was performed for the null hypothesis that the fraction of *B. anynana *UniGenes in each GO category relative to the total number of *B. anynana *UniGenes with an assigned category is equivalent to the fraction of *D. melanogaster *genes in that category relative to all *D. melanogaster *genes with a GO assignment. For the cases where Chi-square p > 0.0001, p-values are provided. All other cases are p < 0.0001.

### DNA sequence polymorphisms and microsatellite repeats

We identified a total of 14,163 SNPs (of any minor allele frequency) in 1,111 of our 1,257 non-singleton UniGenes (Figure 1). We also counted the number of "double-hit" SNPs (SNPs with a minor allele count of *at least *two; see Methods). These SNPs are much less likely to be errors generated during library construction than "single-hit" SNPs but they can only be identified from alignments with a depth of at least four ESTs. We observed a total of 2,222 double hit SNPs. Of our 450 UniGenes of alignment depth four or greater 316 (~70%) had at least one "double-hit" SNP and, of these, 272 (~86%) had a significant BLAST hit to at least one of the fields in Table 3. That is, UniGenes with double-hit SNPs are somewhat highly expressed and as a result are likely to have some associated annotation.

We identified 320 di-, tri-, tetra- and penta-nucleotide repeat microsatellites in our UniGenes (Table 5) with minimum repeat thresholds described in the Methods. Of the identified microsatellites, 73 (23%) were associated with sequence alignments greater than one, and of these, ten (14%) were polymorphic in our sample, having up to seven segregating alleles. Details about the microsatellites identified are provided in Table S6 [see Additional file 6] and include the repeat sequence, the number of times it is repeated, and its location within the host contig sequence.

**Table 5:** Microsatellites found in wing UniGenes

Repeat size	Repeat nr	Total	Non AT^a^	Aligned^b^	Polymorphic^c^
2	>5	26	12	5	2
3	>3	243	129	56	4
4	>3	44	24	12	4
5	>3	7	5	0	0

^a ^Microsatellites whose repeat unit is not composed exclusively of the nucleotides A and T, ^b ^Total numberof microsatellites occurring in a region where the alignment depth is greater than one, ^c ^Total numberof microsatellites for which we observed more than one allele in our EST collection. Details in Table S6 [see Additional file 6].

### *B. anynana *EST database

Apart from all ESTs being deposited in GenBank (Table 1) and, from there, downloaded into ButterflyBase [37], our *B. anynana *EST database is available in two complementary formats. A large flat file [see Additional file 4] suitable for importation into a local database program and a web-accessible openSputnik version [53]. The openSputnik version contains a great deal of information for every EST and every contig and is of the greatest utility for obtaining much information about a few sequences. The downloadable flat file, on the other hand, is useful for typical simple queries of many contigs of special interest. The fields in the flat file include, for each contig: the consensus sequence and sequence length the predicted protein, the total number and a list of all constituent ESTs, the best BLAST hit with E-value lower than 1e-05 (including gene name, E-value, frame in the case of a BLASTX, and URL to that gene's description where available) over several gene collections of interest (*cf*. Table 3). In the case of BLAST against the *B. mori *whole genome collections, we also provide information about the gene location (scaffold and nucleotide position within). The flat file also includes "hot links" to the alignments for every non-singleton contig. The openSputnik database, on the other hand, has extra information available, including the ESTs flagged as low quality and excluded from the analysis in this report, a number of supplementary BLAST fields, and UniGene functional annotations based on mappings of Swissport and InterPro to GO [54].

## Discussion

We carried out an EST project designed to identify a large number of novel *B. anynana *genes and additionally identify SNPs in a fraction of those genes. We biased gene discovery towards genes expressed in wing tissue during the developmental stages when patterns on the wings of butterflies are known to be determined (Table 1). Sequencing of ~10,000 PCR-amplified clone inserts was carried out in two rounds, so that highly redundant ESTs identified during the first round could be used in a library-subtraction scheme aimed at minimizing total EST redundancy. Assembly of 9,903 ESTs allowed us to identify 4,251 *B. anynana *wing disc UniGenes, of which 2,461 were annotated based on BLAST analyses against gene collections available for other organisms (Table 3). Our alignments of different natural alleles allowed us to identify SNPs in many multi-member UniGene contigs (Figure 1). Finally, we identified 320 potential microsatellites markers in expressed sequences, with about 23% located in alignments of depth two or greater, and 14% of these being polymorphic. Overall, our project has generated genomic resources that will be invaluable for exploring the potential of *B. anynana *as a model in ecological, evolutionary, and developmental genetics.

### Sequence ESTs from 3' end to facilitate combined gene and SNP discovery

Using a modified 3' adaptor and modified 3' primer (see Methods), we introduced "point mutations" in the polyA tail of the cDNA clones to facilitate sequencing through this repetitive region. A 3' sequencing effort results mostly in information about the 3' untranslated regions (UTRs) of genes, which despite their role in mRNA processing [55,56], are often not highly conserved and thus do not BLAST 3' UTRs of other organisms. However, for genes which have a relatively short 3' UTR, part of the EST sequence is coding region, allowing for gene identification in many instances. The average size of 3' UTRs in invertebrates is estimated to be ~300 bp [57], which is shorter than the average size of our UniGenes (583 bp, Table 2), but there is much variation around this length. In our project, 48% of the assembled UniGenes did not have a predicted peptide (Table 2) suggesting a 3' UTR longer than the sequence read.

Despite the difficulties of gene annotation associated with 3' UTR reads, the advantages are many-fold. The likelihood of overestimating the number of UniGenes is higher in 5' projects relative to 3' projects, as cDNA clones are often not full length and 5' sequencing can result in the identification of non-overlapping parts of the same gene. In contrast, except for cases of alternative 3' UTRs or polyadenylation sites [58], all EST sequences for a given UniGene in a 3' project share a common poly-A tail and generally align. The resulting alignment depths, which are greater than those typically seen in 5' projects, together with our deliberate use of outbred individuals for building the cDNA libraries (Table 1), enabled us to identify SNPs in many of our UniGenes (Figure 1). Finally, the 3' ends of genes are better features for expression arrays, since probe labeling for gene expression profiling is typically 3' biased and cross-hybridization amongst gene family members is minimized as 3' UTRs are generally less conserved than coding regions.

### Identifying genes expressed in developing butterfly wings

Our library subtraction protocol enabled us to reduce the redundancy typical of EST projects so as to maximize the number of new genes identified and still have alignments deep enough for polymorphism identification. We have identified 4,251 *B. anynana *wing UniGenes of which 2,461 (~58%) had a BLAST hit against at least one of the genomic collections in our analysis (Table 3).

Although it is possible that the 1,790 genes with no BLAST hits to any of the collections in Table 3 are unique to butterflies (or *B. anynana*), it seems more likely that these UniGenes have read lengths shorter than the length of the 3' UTR (see above) and that the UTR is not conserved to *B. mori *(the model lepidopteran organism) or to any sequence in the lepidopteran collection (Figure 2 and Table 3). About 74% (1,324) of the UniGenes with no significant BLAST hit in our analysis had no corresponding predicted protein. Conversely, only 15% of the UniGenes with predicted proteins failed to have significant BLAST hits. A collection of 5' reads for our UniGenes and the growing gene collections available for butterflies will likely help in identifying additional orthologs.

The closest organism to *B. anynana *with extensive genomic collections is the moth *B. mori*, but moths and butterflies have diverged more than 70 million years ago [59] and it is conceivable that even coding regions might not be conserved over such evolutionary distances (*e.g*., [60]). Furthermore, the sometimes spectacular differences between butterflies and moths in morphology (*e.g*., color vision and derived wing patterns in butterflies) and behavior (*e.g*., diurnal lifestyle) also argue for the possibility of there being genes in each group with no obvious homolog in the other. In fact, of the 2,202 peptides predicted from our *B. anynana *contigs, 215 (~10%) with predicted peptides longer than 100 amino acids had no significant BLAST hit to any database. Whereas these peptides may represent artifacts from alignment and peptide prediction algorithms, it is also possible that they correspond to new or highly diverged proteins in butterflies. It will be interesting in future work to determine whether these genes have expression patterns suggestive of a role in wing color patterning and if they are seen in other butterflies.

*Heliconius *butterflies are phylogenetically closer to *B. anynana *(both are nymphalids) than *B. mori*, and have rapidly growing gene collections. All *Heliconius *genes deposited in the standard databases at the time of analysis were included in our lepidopteran and invertebrate collections (see Methods) for gene identification based on sequence homology (Table 3), but not singled-out for a separate BLAST analysis. Both *Heliconius *and *B. anynana *collections are incomplete in terms of UniGene number (~4,200 for *B. anynana *as reported here or ~5,700 as assembled in ButterflyBase, and ~3,700 for *Heliconius *deposited in ButterflyBase; [37])) and their length (the vast majority of UniGenes are not full length and whilst *B. anynana *ESTs were sequenced from the 3' end, those of *Heliconius *are mostly 5'end reads) and thus a separate analysis is of limited value for gene identification. The comparison between these nymphalid collections will become much more valuable as both get larger (see [61])

### Gene annotation via homology with *D. melanogaster*

*D. melanogaster *is the insect with the largest mass of available information on genome sequence and its functional characterization. Using these resources can be of great value for gene identification and characterization of new insect genomic collections (Table 4). We were able to attempt a functional annotation of our *B. anynana *contigs that BLAST *Drosophila *either directly or indirectly via *Bombyx*. Three of the classes of genes annotated based on GO classifications for *Drosophila *deserve special reference.

The first class is that of the so-called *house-keeping *genes which included 120 structural constituents of ribosomes (*e.g., RpS13*, contig 633 in Table S4 additional file 4), and 4 tubulin genes (*e.g., αTub84B*, contig 860), as well as many other genes characteristically expressed at high levels in many cell types. Given that such genes are likely to be discovered in essentially all EST projects, they may turn out to be a valuable resource for comparative studies. Sequence comparisons across lepidopteran species can provide insight into the molecular evolution of particular gene families, and might be useful to build a time-calibrated phylogeny for higher lepidopteran taxa [62]. Furthermore, if genes belonging to these classes are added to genetic maps for several lepidopteran species, we will be able to further analyze and hopefully confirm the patterns of synteny in this group (see [63]). Comparing gene order using traditional linkage mapping-based approaches is likely to remain the preferred method of synteny inference in lepidopterans for some time, as lepidopteran genomes are large by insect standards (often > 400 Mb) and whole genome shotgun sequencing projects [34,35] have thus far been difficult to assemble into scaffolds large enough for chromosome assignment or even small-scale synteny analysis.

The second class of genes worth highlighting includes those known to be involved in fly wing development, which are *a priori *candidates for contributing to wing pattern formation and variation (*e.g., apterous*, contig 3182, and *shot*, contig 4279). Table 4 lists a number of genetic developmental pathways in this category, including 21 genes from fly wing disc development, 13 from tracheal system development, and genes from different signaling pathways involved in wing development (*e.g., Wnt *and *Notch *pathways, represented for example by *nejire*, contig 4401, and *Notchless*, contig 1312). The availability of sequences for such genes will enable segregation tests to assess their involvement in pattern development and contribution to pattern variation within and between species (*c.f*. [19,30]).

Finally, a third and exciting class of genes we identified includes those not characteristically expressed in *Drosophila *wing imaginal discs, which seem to have been recruited to function during butterfly wing development. Our *B. anynana *wing ESTs include 4 genes (Table 4) known to be involved in eye pigmentation in *D. melanogaster *(*e.g., Henna*, contig 4143, and *vermillion*, contig 406), which likely have been recruited to produce butterfly wing pigmentation [19,64,65]. This discovery illustrates a limitation of generating butterfly wing candidate genes based solely on *D. melanogaster *wing development. Furthermore, it provides yet another exciting example of the possible co-option of insect-wide developmental pathways to produce butterfly-specific traits. Wings covered with colorful scales are a morphological novelty in the Lepidoptera whose development has been shown to involve a number of genetic pathways known from *D. melanogaster*: Formation of particular wing pattern elements relies on pathways involved in overall wing formation in *Drosophila *[15,26-29], and wing scale production recruits a pathway from *Drosophila *bristle development [66]. Our results illustrate a third potential example of genetic co-option on butterfly wings, with known pigment genes used in the *Drosophila *eye also used to color wings (see also [19,65]).

### Sequence polymorphisms in *B. anynana *wing genes

Sequence quality is of fundamental importance for accurate SNP identification. Historically, DNA sequences associated with EST projects have not included quality scores. We used lowercase letters for bases with PHRED scores [67] lower than 20 (*i.e*. the probability that an uppercase, high-quality base is called wrong is less than 1% [68]) and ignored "low quality" bases for SNP analysis. Figure 1 shows that we have identified a large number of SNPs [see details in Additional file 5]. In fact, all UniGene clusters with an alignment of depth of six or greater have at least one SNP. Different explanations can be put forward to explain this high number. First, there were several steps where errors could have been introduced during library construction and amplification, such as the use of reverse transcriptase during first strand synthesis, or the 22-cycle exponential amplification of the reverse-transcribed products carried out prior to cloning (see Methods). Such errors would not necessarily result in low quality bases and could potentially be identified as SNPs. Secondly, assembly and alignment algorithms can both over-assemble and misalign, especially when the sequences being studied are known to harbor SNPs. Incorrect alignments, combined with possible instances of pseudogenes or gene duplications in our EST collection will falsely increase estimates of polymorphism level. Finally, it is possible that our outbred source population did have polymorphism levels approaching those observed. The levels we observed are only approximately twice as high as those generally seen in well-studied populations of *D. melanogaster *(*c.f*. [69]), and not inconsistent with the only previous SNP survey in *B. anynana *[30].

As discussed above, it seems likely that a substantial fraction of the over 14 thousand SNPs identified in 1,111 of our non-singleton UniGenes (Figure 1) might not be actual segregating polymorphisms. A conservative estimate of the number of "reliable" SNPs can be made by only considering "double-hit" SNPs (see Methods) which are far less likely to be associated with the unreplicable random errors arising from library construction and clone amplification. A total of 2,222 "double-hit" SNPs were identified in 316 of our 450 UniGenes with an alignment depth of four or greater. Although many of the 11,941 SNPs with a minor allele count equal to one may be true SNPs, this class is undoubtedly enriched for erroneously identified sequence polymorphisms. Furthermore, even if real, SNPs with an observed minor allele count of one are of questionable utility, as population genetics theory predicts their expected population frequency to be much lower than 1/*d*, where *d *is the depth of the alignment [70].

### Developing genomic resources for *B. anynana*

The genes and SNPs we identified will be the basis for the development of important genomic tools for *B. anynana*, which will be invaluable in the effort to understand the genetic basis of variation in butterfly wing patterns and the evolutionary and developmental processes shaping that variation.

The DNA sequence polymorphisms identified in our study will be used to build a high-density linkage map in which the markers are SNPs in genes expressed in developing wings. Such a map will have added value relative to a map constructed entirely of anonymous markers, as i) mapping QTLs to individual genes is often only possible by testing candidate genes in the interval to which a QTL is mapped (*cf*. [71-74]), ii) testing polymorphic markers in candidate genes for co-segregation with a character of interest is an efficient way to be able to exclude those genes as contributing to variation in the character, and iii) it is possible that synteny among lepidopterans (see [63]) can be used to suggest candidate genes for any QTL mapped to an interval between two gene-based markers in maps of different species. Although we have identified "reliable" SNPs in only a subset of our UniGenes (and not necessarily those most interesting from the point of view of development), we now have gene sequence tags for many developmental candidate genes in which we can develop markers and determine the relative position on a linkage map for the 28 chromosomes of *B. anynana *. We have also identified 320 microsatellite repeats (Table 5) which can be tested for polymorphism levels in outbred *B. anynana*, and hopefully add to the 28 polymorphic microsatellite loci already characterized in this species [40]. Microsatellites markers have been difficult to develop in lepidopterans based on traditional methods of screening gDNA libraries [75] (but see [76,77]). Modest repeat number microsatellites potentially located within coding regions, such as those we identified, are likely to be somewhat less polymorphic than gDNA-based microsatellites. However, markers associated to expressed sequences are more likely to be useful in other species and in the context of comparative mapping.

PCR amplicons corresponding to our UniGene set can be used to generate high-density gene arrays to monitor changes in gene expression levels associated with different types of changes in wing pattern phenotype (*e.g*., from artificially selected lines, mutant stocks, or sections of wing discs that fate-map to different adult pattern elements) [19]. Targeting 3' UTR sequences has important benefits in relation to creating high-density arrays and future gene expression experiments. Untranslated regions diverge very quickly, even within gene families, and thus cross-hybridization among features on the gene arrays is less of an issue than when features correspond to protein coding regions. Moreover, as most current methods of labeling polyA RNA for gene expression analysis show a marked drop off in efficiency with distance from the polyA tail, array features corresponding to 3' UTRs are more likely to show above background signals after hybridization with labeled RNA.

### Genomic resources for Lepidoptera

The EST resource described here represents the most extensive collection available for any butterfly species. Butterflies and moths comprise the order of Lepidoptera which is a very species-rich order of holometabolous insects and includes many organisms of great economical (*e.g*., agricultural pests) and educational (*e.g*., butterflies have long been favorite examples in efforts of stimulating public understanding of science) value. However, genomic resources for Lepidoptera are scarce relative their biological and societal importance. The vast majority of gene sequence information available for this group come from recent releases of the silkmoth EST [32,33] and genome projects [34,35]. Just prior to submission of our ESTs to GenBank (March 2006), this reference database had about 131,000 ESTs from *B. mori *and the rest of all lepidopterans represented by only about 21,000 ESTs. Of these, a little over 10,000 were from Papilionoidea (the "true butterflies") and mostly came from species of the genus *Heliconius *whose collection is still growing. On-going projects to develop genomic resources in these and other butterfly species, together with the organized effort of inventorying such resources in a dedicated database (ButterflyBase; [37]) will enable exciting comparative analysis and progress in making the most of this group of organisms as study targets in various areas of biological research. EST projects, such as the one described here are, in the short term, likely to remain the best approach to genomic analysis in butterflies whose relatively large genome size (around 400 Mb) renders genome projects expensive and difficult to assemble.

## Conclusion

Color patterns on butterfly wings have already made key contributions to the effort of integrating evolutionary and developmental biology in the study of adaptive morphological evolution. Wings patterns are highly diverse, yet structurally relatively simple (a single cell layer), ecologically relevant, and suited for many levels of genetic and developmental dissection. Scarcity of genomic resources in this group of organisms has slowed the deepening of our detailed understanding of the genetic and developmental mechanisms that underlie the production of diversity in wing patterns. By targeting cDNA from developing wings at the stages when pattern is specified, the EST project described here biased gene discovery towards genes potentially involved in pattern formation. Assembly of 9,903 ESTs from a subtracted library allowed us to identify 4,251 putative gene objects including 2,461 with homologs in publicly available gene collections, and over 200 potentially representing novel or highly diverged butterfly genes. Analysis of putative gene function through Gene Ontology databases identified different classes of genes, including "housekeeping" genes recurrent in EST projects, classical wing development genes and also new candidate genes which are typically not associated with wing development in the genetic model *D. melanogaster*. By targeting cDNA from a large number of outbred butterflies (ensuring representation of different alleles) and sequencing ESTs from the 3' end (maximizing alignment depths), our project combined gene discovery with identification of putative markers (including SNPs and microsatellites) in expressed genes.

The genetic resources stemming from the EST project described here can be used to pursue a detailed understanding of the genetic basis of phenotypic variation, as well as address fundamental questions in evolutionary biology. Identified ESTs will aid in the identification of the specific genes, gene regions, and possibly nucleotides underlying phenotypic variation of adaptive traits. The color patterns on *B. anynana *wings are well suited for an analysis of different modes of phenotypic variation, enabling the comparison between the basis of variation within and across species, between standing quantitative variation and mutants of large effect, and of variation generated by developmental plasticity in relation to environmental cues [19]. This variation can, furthermore, be explored at different levels of biological organization, ranging from the genetic pathways involved in trait formation [10] to the ecological relevance of pattern variants [78-80]. This system provides an excellent opportunity to address other key issues in evolutionary-developmental biology, including the evolution of morphological innovations (as are butterfly wing color patterns) and the co-option of existing developmental pathways to produce such new phenotypes.

## Methods

### Butterfly developing wings

Wing discs were dissected from developing butterflies sampled from a large (minimum of 400 adults each generation) outbred stock of *B. anynana *([40,81,82]) maintained in the laboratory at the University of Leiden and used in a series of artificial selection experiments to produce divergent wing phenotypes [21-23,83,84].

We dissected both fore- and hindwing discs from developing butterflies at 5 different stages: 5^th ^instar larva (5TH), crawler (when the 5^th ^instar larvae stops feeding and crawls up the host plant to pupate) and pre-pupae (CPP), and male and female pupae at 24 h (24H), 48 h (48H), and 72 h (72H) after pupation (Table 1). Dissections were carried out in cold PBS, and dissected wing discs were immediately placed in RNAlater (AMBION) and stored following the manufacturer's recommendations until RNA extraction.

### Wing cDNA libraries

Total RNA was isolated using TriZol reagent (INVITROGEN), quantified in a spectrophotometer, and its quality verified in agarose gel. cDNA libraries were created using CLONTECH's SMART technology (TAKARA) which selectively transcribes and amplifies full-length polyadenylated mRNA, following the manufacturer's recommendations except where noted. We used ~900 ng of total RNA as starting material from each wing collection. In order to increase the efficiency of 3'-end sequencing directly from PCR-amplified cDNA clones, we used a modified 3' 54-mer adaptor (ATTCTAGAGACCGAGGCGGCCGACATG(T)_4_G(T)_9_C(T)_10_VN) for the first-strand synthesis, and a modified 3' primer (ATTCTAGAGGCCGAGGCGGCCGACATG(T)_4_GTC(T)_4_GTTCTGTTTC(T)_4_VN) for the cDNA amplification step. These modified oligonucleotides effectively convert the long run of adenosine residues into a sequence that causes fewer problems for dideoxy sequencing chemistries. Following first strand synthesis, the SMART technology allows for exponential amplification of polyA containing cDNA; we carried out 22 such cycles. We obtained RNA and amplified cDNA separately for each of the 5 stages of development, for both hind- and forewings, and for male and female pupae, but pooled wing discs from different individuals within each stage/wing/sex prior to RNA isolation. Within-stage amplified cDNAs were pooled in equimolar amounts (as estimated from agarose gels) before the DNA digestion step that precedes insert ligation into the λTriplEx2 phage arms. Phage were packaged using Packagene Lambda DNA (PROMEGA) and all libraries were amplified from ~10^6 ^initial pfus to final titers ranging from 10^9 ^to 10^10 ^pfu/ml, following CLONTECH's library protocol. Libraries were mass excised to pTriplEx2 and grown in *E. coli *strain BM25.8 (CLONTECH). Individual colonies were picked into 50μl of 0.01% Tritron-X. One μl of this solution was used as template in a 10 μl PCR reaction with 0.25U ExTaq (TAKARA), 4 pmole of each of the primers (forward: CTCGGGAAGCGCGCCATTGTGTTGGT, and reverse ATACGACTCACTATAGGGCGAATTGGCC), and dNTPs and reaction buffer according to the enzyme manufacturer's recommendations. An initial denaturing step of 5 min at 95° was followed by 35 cycles of 30 sec at 95°, 30 sec at 68°, and 3 min at 72°. We picked and amplified DNA from about 4,600 clones from each of the 5 libraries.

### Library subtraction

We sequenced an initial set of ~2,000 PCR-amplified clone inserts (sequencing described below), divided roughly equally between the five libraries. This generated 1,934 high quality sequences which were assembled into 970 UniGenes. 777 of the individual reads assembled into 73 "abundant" UniGenes (defined as those harboring four or more ESTs). We designed ~100 oligonucleotide probes matching these abundant transcripts [see Additional file 1] to use in a library subtraction procedure to minimize EST redundancy. Each oligonucleotide probe was radioactively labeled in 5μl reactions (2.5 pmole of oligo, 5U T4 PNK, and 33 nmol ATP^33 ^(10μCi)) for 45 min at 37°, terminated by heating to 80° for 15 min, and then pooled before removing unincorporated ATP using MicroSpin G-25 columns (AMERSHAM BIOSCIENCES). We used these probes to screen high-density arrays of unsequenced PCR-amplified clone inserts in order to identify transcripts not already sequenced in the initial set of ~2,000 clones. High density arrays were created using half the PCR volume for each amplified clone that was dried down and re-suspended in 5μl of denaturing buffer (0.5 M NaOH, 1.5 M NaCl) and spotted onto Nylon filters (MILLIPORE) as described in [85,86]. For each stage library, we produced one filter containing 4,608 features, including PCR products already sequenced. Duplicate filters were hybridized with three different sets of probes: a pool of oligonucleotides corresponding to transcripts seen six or more times in the initial sequencing, a pool corresponding to transcripts seen four or five times, and, to monitor PCR yield, a control oligonucleotide probe internal to the primers used to amplify clone inserts but common to all inserts (AAAGACAAAACATGTCGGCC). Filters were hybridized, washed, exposed, stripped, and spot intensity measured as in [85,86]. Log-transformed dot intensity for the abundant transcript probes versus control probe [see Additional file 2] were plotted and PCR products corresponding to spots with low intensity measures for the control probe excluded from further analysis. These amplicons represented ~35% of the total and likely corresponded to inserts that were poorly amplified or non-recombinant clones. The remaining spots segregated into two distinct classes: high for the redundant transcript probes relative to the control probe (*i.e*., those already sequenced) and low for the redundant transcript probes relative to the control probe (*i.e*. new inserts). High-yield PCR features corresponding to clones harboring novel inserts represented ~42 % of the total PCR products arrayed.

### EST sequences

We used a QIAGEN BIOROBOT to "cherry-pick" 8,832 PCR products harboring putative novel inserts identified based on the subtraction procedure described above. We outsourced sequencing of the 3' ends of the ESTs to GENAISSANCE PHARMACEUTICALS (NEW HAVEN, CT, USA). In total, we sequenced ~2,000 preliminary and 8,832 post-subtraction PCR-amplified clone inserts using the sequencing primer GGCCAAGTGAGCTCGAA. Quality scores were assigned to the sequence traces using PHRED [67]. EST assembly and annotation engines typically ignore quality scores, so we wrote a custom PERL script that uses lower- and upper-case letters to categorize basecalls into low (PHRED<20) and high (PHRED> = 20) quality bases, respectively. This PERL script allowed us to collapse basecalls and their respective quality scores to a single fasta file with case used to designate base quality. Case was preserved throughout our subsequent database curation efforts, and is available in Table S3 [see Additional file 3] together with other information for each EST (*e.g*., length, tissue source).

### Sequence processing and assembly

The EST sequences were clustered and assembled within the openSputnik EST analysis pipeline [52]. Prior to assembly, regions of low-complexity were masked using RepeatBeater (BIOMAX Informatics, Martinsried, Germany) and vector remnants were removed using CrossMatch with the UniVec database [87] modified to include the specific polylinkers we used in the cloning process. Clustering was performed using the Hashed Position Tree (HPT) algorithm (BIOMAX Informatics). An HPT similarity link threshold of 0.7 and maximum distance of six steps was imposed to define a cluster within the similarity network, thus encouraging the separation of likely paralogs. Multi-member clusters were assembled into UniGenes and singletons using the CAP3 method with default settings [88].

Consensus nucleotides for polymorphic sites were assigned based on majority-rule nucleotide frequency (*e.g*., in an alignment of depth three, if one sequence is an A and two are C, the consensus will be C). The consensus sequence for each assembled UniGene was assigned a complexity score representing the linguistic complexity of the oligonucleotide "words" that build the sequence, calculated using the "complex" method from version 2.2.0 of the EMBOSS package [89]. "Complex" was run using a window size of 25 nucleotides and a window displacement step of five nucleotides and considered all oligonucleotides between four and six residues in length. UniGenes with complexity scores lower than 0.49 were removed from the analysis. The consensus sequences and associated complexity scores for all contigs, as well as other sequence characterization parameters (*e.g*., sequence length and total number of ESTs in each contig) are provided in Table S4 [see Additional file 4].

For each cluster's consensus, we derived likely protein coding sequences by parsing the best BLASTX match and filtering the results using the arbitrary expectation value of 1e-10. The derived candidate coding sequence was used to train the ESTScan method [90] for properties and nuances of *B. anynana *codon usage. ESTScan was run using default settings with the derived *B. anynana *model to predict coding sequence across the whole sequence collection. The best BLASTX match to the SwissProt database [91] was used as the sequence reference. Peptide sequences of length equal or greater than 20 aminoacids were derived for *B. anynana *and used as the basic scaffold for peptide-based annotations in openSputnik [52]

### Annotation: gene identification

The cluster consensus nucleotide sequences and predicted peptide sequences were assigned likely identities within the openSputnik [52] pipeline using BLASTN (fields ending in ".nuc" in Table 3) and BLASTX (fields ending in ".pro" in Table 3) [92]. Sequence comparisons between *B. anynana *and other insect sequence collections included all *D. melanogaster *genes in FlyBase ("Dmel.nuc" with 20,016 genes, and "Dmel.pro" with 19,369 peptides; BDGP4), from *Bombyx mori*, the ESTs [32,33] (116,541 entries retrieved from EMBL EST database and assembled in openSputnik [52]into 26,089 clusters and 12,904 predicted peptides [93]) and the published Whole Genome Shotgun (WGS) sequences [34,35], and all lepidopteran sequences in EMBL release 83, excluding the ones from *Bombyx mori *mentioned above ("lep.nuc" with 40,501 entries). The *B. mori *collections were organized as follows: hits to "Bmori.nuc" correspond to the best BLAST hit to nucleotide sequences of the openSputnik-clustered EST collections [32,33] or the predicted coding sequences from one of the WGS projects [35], hits to "Bmori.pro" correspond to the best BLAST to peptide sequences of the openSputnik-clustered EST [32,33] or the predicted peptides from one of the WGS projects [35], and finally hits to "Bmori.wgs.nuc" correspond to best BLAST to one or both of the WGS collections [34,35]. Homology comparisons against the Rfam database [94] were used to identify candidate structural and non-coding RNAs ("Rfam.nuc"; 34,496 sequences from Rfam 6.0), and comparisons against the collection of organellar DNA were used to identify non-nuclear genes ("organel.nuc"; 67,548 sequences downloaded EMBL in April 2004). BLAST analysis against the whole genome of *E. coli *K12 (from NCBI) was used to identify potential bacterial contaminants ("Ecoli.nuc). Further annotation was accomplished using BLASTX against a number of peptide collections (Table 3); "NonRed.pro" (a non-redundant protein sequence database constructed from all TrEMBL, all SwissProt, and all PIR sequences; 2,268,590 entries), "invert.pro" (all TrEMBL protein sequences from invertebrate species; for a total of 303,441 peptide elements), "plant.pro" (EMBL release 83, 195,673 protein sequences from plant species), and "Swiss.pro" (SwissProt database release 48.1 with 196,679 sequences). All BLAST-based homology results were filtered using the expectation value of 1e-05.

Some BLAST fields were combined for a clearer summary of the gene identification effort (Table 3, Figure 2). The field "Dmel" in Figure 2 corresponds to all those contigs with a significant BLAST result for at least one of the fields "Dmel.nuc" and "Dmel.pro". In the same way, all *B. mori *EST- and WGS-derived fields were combined to account for all significant BLAST results against the *B. mori *collections ("Bmori"), "invert.pro" and "lep.nuc" were combined into the "InvLep" field, and "organel.nuc" and "Rfam.nuc" were combined into the "nonnuc" field for contigs showing best significant hits to non-nuclear or non-protein coding gene collections (Table 3). Finally, sequences were assessed for possible contamination by searching for homology to the *Escherichia coli *genome ("Ecoli.nuc", possibly from bacteria living inside dissected larvae and adults or used during the cloning process) and to plant collections ("plant.pro", possibly from plant material from the larvae gut). We considered potential contaminants UniGenes meeting the following criteria: 1) the best BLAST hit (lowest E-value) was obtained in the analysis against "plant.pro" or "Ecol.nuc", and 2) the best hit to any invertebrate-specific field ("Dmel", "Bmori", "InvLep") was at least 100 fold higher to insure they were not simply highly conserved genes.

### Annotation: functional and phylogenetic context

The openSputnik database contains a number of fields pertaining to the functional annotation of the genes identified. These include domain analyses, which were performed using the InterPro software [95], and functional and role assignments which were performed using SwissProt to GO and InterPro to GO mappings [54].

Further functional assignments, using gene identification obtained via the two most relevant genomic collections (those of *D. melanogaster *and *B. mori*) were performed using the software GOminer [50]. Based on GO classifications available for *D. melanogaster*, GOminer assigns GO categories to the list of *D. melanogaster *CG numbers for which we found homologous genes among our UniGenes (either directly, based on the BLAST analysis against the *Drosophila*-specific fields, or indirectly, based on the BLAST analysis against *B. mori *ESTs annotated based on the *D. melanogaster *gene collection). The latter group corresponds to *B. anynana *genes which do not have a significant BLAST hit to the *D. melanogaster *collection, but do have a significant hit (E-value below set threshold of 1e-05) to a contig from the *B. mori *EST collection which, in turn, had a significant hit (E-value below 1e-05) to a *D. melanogaster *CG number.

### Polymorphic marker identification

In order to characterize the SNPs segregating in our samples, we used our multifasta UniGene alignments and a custom PERL script to process them. For each non-singleton UniGene, all ESTs in the alignment were made the same length by padding with "N"s where necessary, and all lowercase (PHRED<20) basecalls were replaced with "n"s (multifasta alignment files available at [49]). In this way, we minimized the impact of low-quality bases on SNP identification. For each UniGene alignment our PERL script produced a new consensus sequence by ignoring N and n and returning a IUB code for polymorphic sites (*e.g*., in an alignment of depth three, if one sequence is an A and two are C, the consensus will be M). The consensus sequences thus produced were analyzed and the total number of SNPs counted. Since ESTs often contain errors that are believed to originate during the reverse transcription step required to make cDNA libraries, we repeated our SNP identification procedure by considering only SNPs having a minor allele count of *at least *two. Such "double-hit" SNPs have proven to be of much greater practical utility than "single-hit" SNPs (SNPs with a minor allele count of *at least *one) in the human HAPMAP project [96].

We used another custom PERL script to identify microsatellite repeats within majority-rule consensus sequences. The script was written to locate di-nucleotide units repeated at least six times, and tri-, tetra- and penta-nucleotide units repeated at least four times.

## Authors' contributions

PB participated in the conception and design of the study, carried out the laboratory work and data analysis and drafted the manuscript. SR wrote and ran the EST processing pipeline, including EST assembly and UniGene annotation, and is responsible for the web-available database. JDG participated in database analysis including expertise in MySQL and PERL. ADL participated in the conception, design, and coordination of the study, including data collection and statistical analysis, and helped to draft the manuscript. All authors read, commented and approved the final manuscript.

## Supplementary Material

Additional File 1 **Table S1**. List of oligonucleotide probes used in the library subtraction process (tab-delimited .txt file; content details in Additional file 8) Click here for file

Additional File 2 **Table S2**. Spot intensities and library subtraction (tab-delimited .txt file; content details in Additional file 8) Click here for file

Additional File 3 **Table S3**. ESTs – flat file for importation in db program (tab-delimited .txt file; content details in Additional file 8) Click here for file

Additional File 4 **Table S4**. Contigs – flat file for importation in db program (tab-delimited .txt file; content details in Additional file 8) Click here for file

Additional File 5 **Table S5**. SNP data from alignments (tab-delimited .txt file; content details in Additional file 8) Click here for file

Additional File 6 **Table S6**. Microsatellite repeats (tab-delimited .txt file; content details in Additional file 8) Click here for file

Additional File 7 **Table S7**. GOMiner summary table (tab-delimited .txt file; content details in Additional file 8) Click here for file

Additional File 8 readme.txt. Details of contents of additional files 1, 2, 3, 4, 5, 6, 7 (tab-delimited .txt file) Click here for file
